# Performance and adaptability comparison between medical students and experienced neurosurgeons using a robotic exoscope with a head-mounted display

**DOI:** 10.1007/s11701-025-02430-1

**Published:** 2025-06-12

**Authors:** Rodrigo Uribe-Pacheco, Anto Abramovic, Matthias Demetz, Aleksandrs Krigers, Raphael Gmeiner, Marlies Bauer, Sara Lener, Daniel Pinggera, Johannes Kerschbaumer, Claudius Thomé, Christian F. Freyschlag

**Affiliations:** 1 Department of Neurosurgery, Medical University of Innsbruck, Anichstraße 35, 6020 Innsbruck, Austria; 2 Department of Neurosurgery, Instituto Nacional de Neurología y Neurocirugía Manuel Velasco Suarez, Mexico City, Mexico

**Keywords:** Exoscope, Robotics, Microsurgery, Learning, Ergonomy

## Abstract

Integrating robotics in the operating room (OR) may revolutionize surgical practice by providing surgeons with enhanced precision, dexterity, and stability. Traditional operative microscopes often lead to significant musculoskeletal discomfort among surgeons due to prolonged, unergonomic postures. The advent of a robot-controlled exoscope (RS) offers a promising solution to these ergonomic challenges by enabling hands-free operation through head gestures. This study aimed to evaluate the usability and ergonomic benefits of the RS by comparing the performance of experienced neurosurgeons to that of students naive to microsurgery. In this study, we included neurosurgeons and medical students who navigated a custom-made parkour after a standardized 30-min training session. Metrics recorded included time to navigate each eyelet, number of commands executed, command errors, technical help required, and the accuracy of centering each eyelet. Post-exercise, participants completed a questionnaire assessing pain, visual quality, usability, and overall satisfaction. Neurosurgeons executed fewer commands (median 40, IQR 30.25–46) than students (median 44.5, IQR 38–57.5, *p* = 0.095). Time taken to navigate the parkour was similar across groups, but neurosurgeons showed greater efficiency from the third eyelet onward. Instances of automatic re-adjustments and reaching the RS physical limits were lower among neurosurgeons. Post-intervention questionnaires revealed no significant differences between groups in terms of comfort, visualization, image quality, depth perception, usability, and pain scores. The RS demonstrated potential ergonomic benefits and usability, with neurosurgeons showing improved efficiency compared to students. The lack of significant differences in post-intervention assessments suggests the RS is user-friendly for both experienced and novice users. These findings support the RS potential to enhance surgical ergonomics and reduce work-related musculoskeletal disorders, though further studies are needed to fully understand the learning curve and long-term benefits.

**Supplementary Information:**

The online version contains supplementary material available at 10.1007/s11701-025-02430-1.

## Introduction

Integrating robotics in the operating room (OR) may revolutionize surgical practice, providing surgeons with unprecedented precision and control. Robotic systems enhance surgical capabilities by providing superior dexterity, stability, and accuracy during complex procedures [1–4], thereby facilitating minimally invasive techniques. These advancements reportedly result in reduced tissue trauma, lower infection rates, and faster patient recovery times [5–7].

Traditionally, operative microscopes require surgeons to maintain a fixed and often unergonomic posture for extended periods due to the necessity of aligning the optic axis from lens to eyepiece. This prolonged use can lead to significant physical strain, resulting in musculoskeletal discomfort, fatigue, and, in some cases, chronic injuries [8–10]. These physical stresses not only affect the immediate well-being and performance of surgeons but also contribute to a higher incidence of work-related sick leave (WRSL) over the long term [11, 12]. The advent of exoscopes offers a promising solution to these ergonomic challenges [13, 14].

The robot-controlled exoscope (RS, RoboticScope; BHS Technologies GmbH, Innsbruck, Austria) projects images from two robot-controlled cameras onto external virtual-reality-like displays (head-mounted display, HMD). The robotic arm is operated hands-free through head gestures guided by an interface. For safety, the RS responds to head gestures only when a foot pedal is pressed, ensuring precise control. This system allows the surgeon to maintain a more comfortable posture without altering the camera position, significantly enhancing ergonomic conditions during procedures [15, 16].

Previous studies demonstrated the ergonomic benefits of the used device, indicating that this innovative device significantly improves ergonomic conditions for surgeons by allowing them to maintain more natural and comfortable postures [17, 18]. This improvement in working conditions could lead to a decrease in work-related musculoskeletal disorders and a reduction in WRSL among neurosurgeons. However, the learning curve associated with this new device remains to be evaluated. As it incorporates novel technology and a different mode of operation than traditional microscopes, understanding how accurately, quickly, and effectively experienced neurosurgeons and novices can adapt to its use is essential.

Our study aimed to evaluate the efficacy by comparing the performance of experienced neurosurgeons on a structured assessment module to that of students who were naive to microsurgery. This comparison provides insights into RS usability and learning curve and highlights its potential to improve neurosurgical procedures’ overall ergonomics and outcome.

## Materials and methods

### Study design

This study aimed to evaluate the usability and ergonomics of the RS in a controlled, standardized setting. Each participant received a standardized 30-min instruction on how to use the device prior to the test. The study involved navigating a custom-made parkour designed to test various commands and tilting movements of the RS. Participants’ movements were filmed throughout the exercise using the RS own camera.

### Participants

Participants included neurosurgeons and voluntary medical students from the authors’ department and medical university. This inclusion allowed for a comparison between individuals highly experienced with conventional microscopes and those with no prior experience. Each participant signed an informed consent form permitting the use of their data and video footage in pseudonymous form. Prior to enrollment, each participant was assigned a study ID for documentation purposes.

### Pre-interventional training

Each participant underwent a standardized 30-min training. This session included instructions on using the foot pedal and HMD to execute commands. Participants’ interpupillary distances and visual impairments were adjusted using dioptric compensation on the HMD.

### Custom-made parkour

**Fig. 1:**
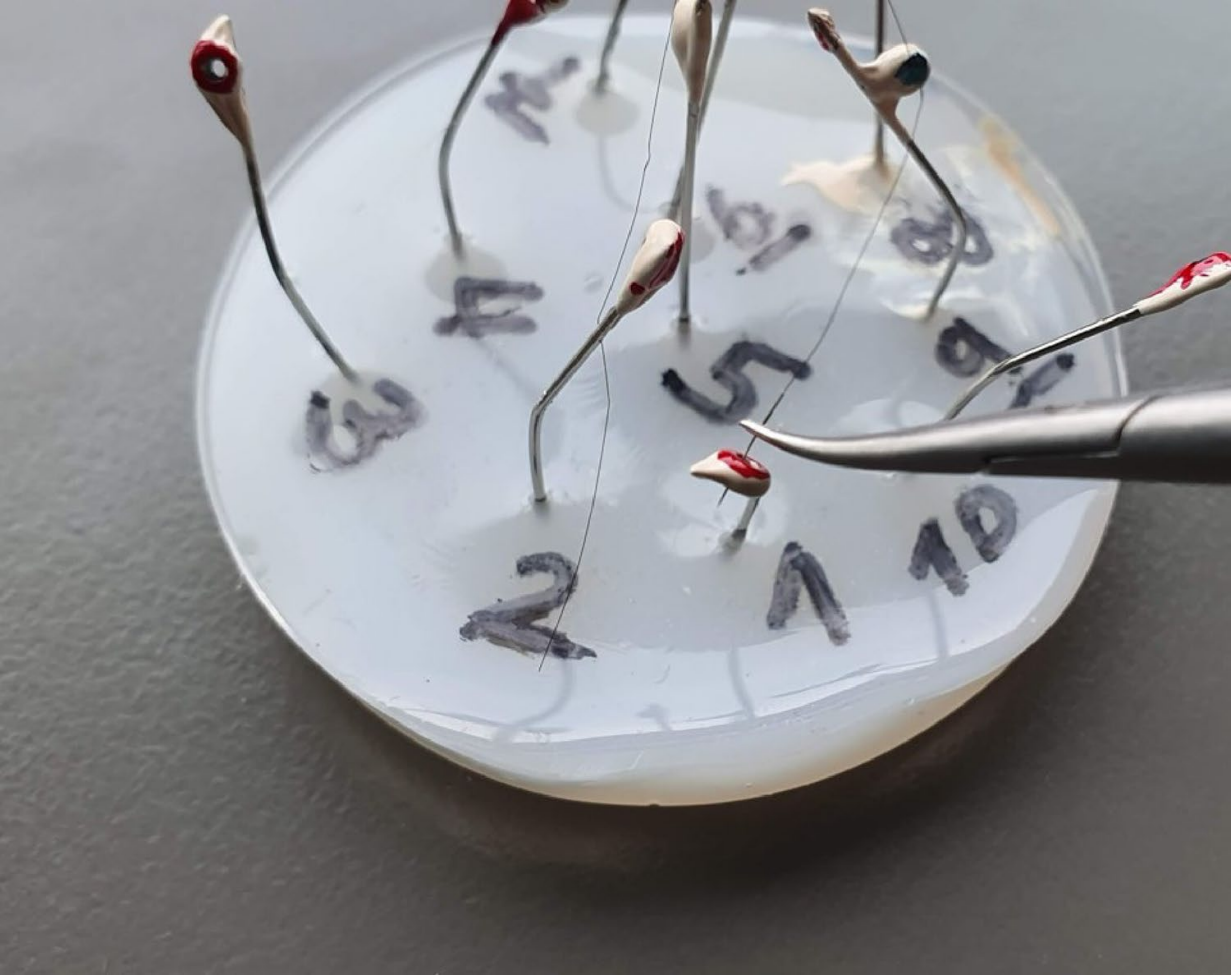
The custom-made microsurgical parkour consisted of ten eyelets that had to be angled and centered in a standardized manner before stitching a 6–0 nylon suture through each eyelet. Throughout the course, time, commands, errors, and accuracy (Bullseye Score) were recorded and evaluated

A custom-made parkour was created using a base plate made of ethylene vinyl acetate (EVA) and metal pins with attached eyelets set at different angles. The parkour contained ten eyelets, each designed to test various tilting movements of the RS. The eyelets were marked with paint and numbered for guidance (Fig. 1).

### Procedure

Participants were instructed to navigate the parkour in a sitting position and in a standardized manner, moving from eyelet 1 to eyelet 10. For each eyelet, the following metrics were recorded:

- Time to navigate each eyelet
- Number of commands executed for each eyelet and in total
- Number of command errors
- Instances of technical help required
- Readjustments of the head-mounted display due to impaired vision
- Reaching the physical limits of tilting of the RS
- Automatic re-adjustments of the RS to achieve the desired angle
- The accuracy of centering each eyelet was assessed using a Bullseye Score ranging from 3 to 30, with 1 being poor, 2 intermediate, and 3 good for each eyelet.

For better understanding of the setup and task execution, a video recording of a key segment of the parkour is provided in the supplementary materials.

### Data collection

Movements were filmed using the RS camera. The video footage was analyzed to document the time required for each eyelet, operating errors, commands per exercise, and commands per eyelet.

### Post-interventional questionnaire

Following the exercise, each participant completed a post-interventional questionnaire assessing head and neck pain, visual quality, security in using the RS, usability, and overall satisfaction with the device.

### Statistical analysis

Statistical analysis was performed using IBM SPSS Statistics, version 25.0 (IBM Corporation, NY, USA). Normal distribution of data was assessed using histograms and the Kolmogorov–Smirnov test. Mann–Whitney *U* test, Chi-square test, or Spearman’s rank correlation coefficient were used to detect significant similarities or differences. A p value of ≤ 0.05 was considered statistically significant.

### Outcome measures

Primary outcome measures included the time to complete the parkour, number of commands, command errors, technical help required, and re-adjustments of the HMD. Secondary outcome measures included the Bullseye Score for accuracy in centering each eyelet, and the number of times the physical limits of tilting were reached or re-adjustments were necessary.

## Results

### Commands

The median number of total commands executed by neurosurgeons was 40 (IQR 30–46), while for medical students, it was 45 (IQR 38–58, *p* = 0.095).

Medical students exhibited significantly more command errors in total compared to neurosurgeons, with a median of 8 errors (IQR 7–11) versus 4 errors (IQR 2–8), respectively (*p* = 0.014). Neurosurgeons generally required fewer commands to navigate each eyelet compared to medical students. For instance, the median number of commands until the fourth eyelet was 3 (IQR 2–5) for neurosurgeons and 6 (IQR 5–10) for students, with a p-value of 0.004. This trend continued with neurosurgeons consistently showing lower median command counts across the second to tenth eyelets.

### Time

**Fig. 2:**
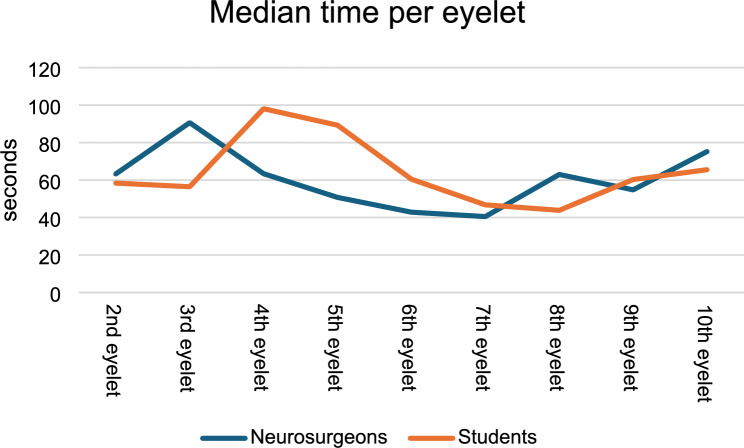
Performance of neurosurgeons (blue) vs. medical students (orange) measured in time needed per eyelet

The time taken to navigate each eyelet showed differing trends. The median time until the second eyelet was 63 s (IQR 40–110) for neurosurgeons, while students took 58 s (IQR 34–82), with a p value of 0.389. This pattern changed after the third eyelet with significantly lower time per eyelet for neurosurgeons. For example, the median time until the third eyelet was 91 s (IQR 63–171) for neurosurgeons, compared to 56 s (IQR 33–81) for students (*p* = 0.005). After eyelet 6, the time taken by neurosurgeons and students to navigate the remaining eyelets showed no significant differences, indicating a convergence in performance between the two groups (Fig. 2).

No significant differences in performance were observed between neurosurgeons of different subspecialties (*p* > 0.05).

Similarly, varying levels of surgical experience within the neurosurgeon group did not lead to significant differences in task performance (*p* > 0.05).

### Questionnaires

The results showed that the median comfort score reported by students was 39/50 (IQR 38–44), while neurosurgeons reported a median of 40/50 (IQR 30–43). Visualization scores were similar between the two groups, with a median of 41.5/50 (IQR 40–48) for students compared to 41/50 (IQR 33–46) for neurosurgeons. The image quality perceived by students had a median of 45/50 (IQR 41–49), whereas neurosurgeons reported a median of 42/50 (IQR 34–47). For depth perception, students scored a median of 34/50 (IQR 30–39) while neurosurgeons reported a median of 39/50 (IQR 32–42). Depth sharpness had a median score of 33/50 (IQR 31–43) among students and 38/50 (IQR 30–43) among neurosurgeons.

In terms of usability, students reported a median score of 33/50 (IQR 26–47) compared to 41/50 (IQR 34–47) for neurosurgeons. Movability scores were slightly higher for neurosurgeons with a median of 36.5 (IQR 29–41), whereas students had a median of 32/50 (IQR 26–37). Training adequacy was perceived similarly by both groups, with students reporting a median of 43/50 (IQR 33–50) and neurosurgeons a median of 46/50 (IQR 35–50). Overall satisfaction showed a median of 44/50 (IQR 39–46) for students and 43/50 (IQR 38–47) for neurosurgeons.

Statistical analysis revealed no significant differences between students and neurosurgeons in most categories, with p values greater than 0.05 in comfort (*p* = 0.878), visualization (*p* = 0.482), image quality (*p* = 0.284), depth perception (*p* = 0.391), depth sharpness (*p* = 0.807), usability (*p* = 0.561), movability (*p* = 0.359), training (*p* = 0.853), overall satisfaction (*p* = 0.854), abdominal pain (*p* = 0.237), lumbar pain (*p* = 0.120), neck pain (*p* = 0.681), upper extremity pain (*p* = 0.399), and lower extremity pain (*p* = 0.473).

### Other metrics

Neurosurgeons had fewer instances of automatic re-adjustments, with a median of 1 (IQR 0–2) compared to 2 (IQR 1–4) for students, though this was not statistically significant (*p* = 0.082). HMD adjustments were minimal for both groups, with neurosurgeons having a median of 0 (IQR 0–1) and students also at 0 (IQR 0–0.25), with a p value of 0.532. Instances of reaching the physical limit of the RS were clearly lower for neurosurgeons, with a median of 3 (IQR 1–5) compared to 5 (IQR 3–12) for students, although no statistically significant difference was noted (*p* = 0.059). Both groups required minimal technical help, with medians of 0, and the difference was not statistically significant (*p* = 0.075). The Bullseye Score, which measured accuracy in centering each eyelet, showed no significant difference between groups, with neurosurgeons achieving a median score of 27 (IQR 24–28) and students 26.5 (IQR 23–28), with a *p* value of 0.973.

## Discussion

Our study evaluated the efficacy of RS by comparing the performance of experienced neurosurgeons with students naïve to microsurgery. Our study found high satisfaction with the RS in both students and experienced neurosurgeons, with comparable results for usability and visualization. In addition, the learning curves were similar between the two groups, indicating that the RS might be beneficial for both experienced neurosurgeons and students naïve to microsurgery. We interpreted that the device is user-friendly and effective across different levels of surgical training and experience.

Several studies have compared training performance and opinions among conventional operative microscopes and exoscopes among trained neurosurgeons [19–21]. However, a comparison among different generations with and without microsurgery expertise must be thoroughly addressed among different exoscope proposals [22]. Considering the exposure among surgical generations towards innovative, virtual-reality, and intraoperative imaging-enhanced modalities is a drift in modern neurosurgery, we consider this aspect is relevant to be addressed since technology-assisted surgery is a growing trend that may be here to stay. Considering the higher incidences of WRSL among microsurgery-performing physicians, finding exoscopes that allow for a more favorable posture compared to the conventional microscope is crucial. Previous studies on the RS have shown its potential to significantly improve ergonomic conditions, highlighting its importance in enhancing surgeon well-being and reducing long-term physical strain [17, 18].

Both neurosurgeons and students demonstrated an acceptable learning curve throughout the trial. Neurosurgeons, with their extensive background in using manual microscopes and performing precise maneuvers, adapted more quickly to the RS. The parkour was intentionally designed with varying distances between the eyelets and different angulations, requiring users to perform a range of movements and adjustments. This design likely contributed to the initially longer task times observed in both groups. However, as both groups showed improved performance over time, a learning curve can be assumed for users regardless of prior microsurgical experience. Neurosurgeons, as shown in Fig. 2, needed the maximum amount of time between eyelet 2 and 3 with an improved time after the third eyelet in the subsequent course. The students, on the other hand, took longer to reach the maximum time between eyelet 3 and 4. This may be because the first 4 eyelets were deliberately designed with more demanding angulations, requiring a higher number of commands and positional adjustments using the RS. This complexity likely explains the increased time needed by both groups to complete these tasks. The subsequent reduction in task duration suggests that users became more familiar with the system, supporting the presence of a learning curve. However, the difference between the two groups could be due to the microsurgical experience of the neurosurgeons. Interestingly, the neurosurgeons showed initial adjustment challenges. However, they adapted more quickly in the following steps, possibly benefiting from their microsurgical experience. In contrast, the students seemed to catch up, which may be attributed to their familiarity with their likely background in video games to effectively interact with the RS interface and controls. This suggests that the RS intuitive design can be advantageous for users with diverse backgrounds, although the learning curve is steeper for those without prior surgical experience.

The study highlighted distinct differences in the trial-and-error process between the two groups. Students performed tasks faster but with more errors and required more assistance compared to neurosurgeons. This is indicative of their lack of experience in surgical scenarios and reliance on trial and error to complete tasks. Neurosurgeons, on the other hand, approached tasks with greater precision and fewer errors [23], reflecting their extensive training and experience in minimizing surgical complications. This underscores the importance of structured training programs to help novices reduce errors as they adapt to not only new technologies. The RS enhanced visualization and control capabilities further augmented their precision, suggesting that experienced surgeons can maximize the benefits of this advanced technology. However, our study found comparable accuracy between the two groups during the parkour, as evidenced by similar bullseye scores. This suggests that despite making more command mistakes, the students were still able to achieve results on par with the experienced neurosurgeons. The comparable performance indicates that the RS’s intuitive design and effective training protocols can bridge the gap between novice and expert users, allowing even those new to microsurgery to reach a high level of accuracy and proficiency in a short amount of time.

Our study found high satisfaction with visualization and comfort using the RS in both experienced neurosurgeons and students, indicating that the RS can be easily used by both groups after a short training period. Both study groups approved the exoscope training prior to the start of the parkour, leading to overall high satisfaction with the device. While the neurosurgical group reported slightly higher satisfaction with usability, likely due to their prior experience with microsurgery, there were no significant differences in adverse events such as lumbar or neck pain. This finding suggests that the RS can be effectively used across all age groups without increasing the risk of musculoskeletal discomfort.

We found very few cases where the participants required a repositioning of the HMD or other technical assistance, similar to the results of the previous results from this study group [17, 18]. This suggests that with thorough pre-interventional training and careful setup before starting the procedure, the RS can be used routinely with minimal disruption. These findings underscore the importance of proper preparation in ensuring smooth and effective use of novel exoscopes in clinical practice. The low incidence of technical issues highlights the robustness and user-friendliness of the RS, making it a reliable tool for both experienced surgeons and those naïve to microsurgery y [15, 17, 24]. This reliability is crucial for integrating new technologies into routine surgical practice, ensuring that the potential ergonomic and operational benefits are consistently realized without significant downtime or complications.

To support a steeper learning curve with the RS, several strategies should be considered, including the implementation of tailored training programs and the use of simulation-based learning environments. These approaches could help both novice and experienced users become familiar with the system more efficiently and reduce the time needed to achieve proficiency. In particular, the use of anatomical training models and cadaver courses—especially when combined with standardized training protocols—may offer a highly effective and structured way to practice and internalize the necessary skills for operating the RS. Beyond neurosurgery, the ergonomic and usability advantages demonstrated in this study suggest that the RS could also be beneficial in other surgical specialties like ENT where precision and prolonged procedures place significant physical demands on the surgeon [15, 16]. The authors thus suggest implementing a short, standardized introduction to the RS interface and command system, followed by a structured training protocol involving tasks with varying angulations and motor demands to promote a steep and effective learning curve.

The RS has the potential to be a valuable tool for preclinical training of students, providing an intuitive and hands-free approach to microsurgical techniques. Using the model from this study, students can practice essential skills in a controlled setting, allowing them to develop precision and familiarity with exoscopic technology before entering the operating room. This could enhance their learning experience and facilitate a smoother transition to clinical practice.

## Limitations

The short duration of the parkour prevented recording of potential side effects that might arise during longer surgical procedures lasting several hours, necessitating additional clinical studies. Furthermore, long-term ergonomic effects could not be fully assessed and warrant further investigation in future studies involving prolonged procedures. The precise number of hours spent on video gaming could not be assessed due to reliance on self-reported data, introducing potential recall bias and variability; however, a greater amount of video game experience may influence participants’ motor skills and thus impact task performance. In addition, participants with limited microsurgical experience may have had difficulty assessing image quality due to the absence of comparisons with conventional microscopes. In sum, there is a need for further studies including direct comparison of the conventional microscope with the RS in a clinical setting. A potential limitation of the RS is that some participants may experience vertigo, as reported in previous studies from this group, which could impact its usability for certain individuals. An interesting avenue for future research would be to repeat one single task multiple times to track individual learning curves and identify potential performance plateaus, which could help optimize training protocols and better understand differences in adaptation based on experience levels.

## Conclusion

The RS demonstrated potential ergonomic benefits and usability, with neurosurgeons showing improved efficiency compared to students. The lack of significant differences in post-intervention assessments suggests the RS is user-friendly for both experienced and naïve users. These findings support the RS’s potential to enhance surgical ergonomics and reduce work-related musculoskeletal disorders, though further studies are needed to fully understand the long-term benefits.

## Supplementary Information

Below is the link to the electronic supplementary material.

Supplementary file1 (MP4 127718 kb)

## Data Availability

No datasets were generated or analysed during the current study.
